# Organo-Montmorillonite (OMMT) Modified SiC/Hydrogenated Epoxy Micro–Nanocomposites for Enhanced Corona Aging Resistance

**DOI:** 10.3390/polym18131662

**Published:** 2026-07-04

**Authors:** Haitao Hu, Hailiang Dong, Mingpeng He, Boxin Ma, Yanli Liu, Junguo Gao

**Affiliations:** 1 Key Laboratory of Engineering Dielectrics and Its Application, Ministry of Education, College of Electrical and Electronic Engineering, Harbin University of Science and Technology, Harbin 150080, China; 2 Dongfang Electric Machinery Co., Ltd., Deyang 618000, China

**Keywords:** micro/nanocomposites, corona resistance, epoxy resin, surface conductivity, conductance nonlinearity, trap characteristics

## Abstract

The concentration of electric fields at the end region of stator bars in large generators can readily induce corona discharge. Under long-term operation, corona discharge may cause drift in the surface conductivity and nonlinear coefficient of anti-corona materials, thereby weakening their capability to homogenize the tangential electric field. In severe cases, this can lead to charring failure of the anti-corona material. To improve the electrical-parameter stability and surface morphological resistance to corona aging of silicon carbide (SiC)-based anti-corona materials under long-term corona exposure, epoxy-resin-based anti-corona materials were investigated in this study. Scanning electron microscopy (SEM) and Fourier-transform infrared spectroscopy (FTIR) were first employed to analyze the effects of corona aging on the microstructure and chemical structure of the anti-corona layer, thereby revealing its failure mechanism. Subsequently, the evolution of surface conductivity, nonlinear coefficient, and surface morphology of bisphenol A epoxy resin (EP)- and hydrogenated bisphenol A epoxy resin (H-EP)-based anti-corona materials during 120 h of corona aging was comparatively investigated. On this basis, different mass fractions of organically modified montmorillonite (OMMT) were introduced into the H-EP-based anti-corona material for synergistic modification. The OMMT used in this study had a particle size of approximately 5 μm and an interlayer spacing of 2.6 nm, and its lamellar morphology and dispersion state in the epoxy matrix were characterized by cross-sectional SEM. Meanwhile, the trap-regulation mechanism of the OMMT-modified anti-corona materials was analyzed using isothermal surface potential decay (ISPD). The results show that erosion of the epoxy resin matrix by corona discharge is the primary cause of internal conductive-pathway disruption and anti-corona layer failure. Compared with the EP-based material, the H-EP-based material exhibited better conductivity and nonlinear stability during aging, although a certain degree of drift still occurred. The incorporation of an appropriate amount of OMMT further improved the corona resistance of the material. Among the investigated samples, the material containing 1 wt% OMMT showed the best performance, with its conductivity stabilized within the range of 10^−13^–10^−11^ S, the lowest variation rate of 104.76%, a relatively stable nonlinear coefficient, and slight surface damage. The ISPD results indicate that the interfaces introduced by OMMT increase the deep-trap density and suppress carrier migration, thereby stabilizing the conductive network. Overall, the synergistic effect of the H-EP matrix and 1 wt% OMMT can effectively enhance the corona resistance of SiC-based anti-corona materials.

## 1. Introduction

With the continuous increase in the installed capacity of large generators and the further deployment of such machines at higher altitudes, the internal electric-field distribution becomes highly nonuniform, making corona discharge more likely to occur at the end region of stator bars [1,2]. Corona discharge on the stator surface continuously erodes and ages anti-corona materials, posing a potential threat to the safe operation of Generators [3,4,5,6,7]. At present, anti-corona materials using SiC as the primary conductive filler exhibit significant fluctuations in both surface conductivity and the nonlinear coefficient during long-term operation as a result of corona aging. These fluctuations intensify electric-field distortion and may lead to a vicious cycle in which nonuniform electric-field distribution and corona discharge mutually reinforce each other, ultimately resulting in insulation aging or even breakdown at the stator-bar end region [8]. Therefore, improving the corona-aging resistance of anti-corona materials and ensuring the long-term stability of anti-corona layer parameters under operating conditions are essential for the reliable operation of large-generator insulation systems and represent an important research direction in this field.

Guangning Wu et al. [9] reported that corona discharge damages materials mainly through four mechanisms: charged ions accelerated by the electric field bombard polymer macromolecular chains at high speed and break molecular bonds; discharge-induced transient local temperature rise leads to localized polymer melting and chemical degradation; partial discharge generates highly reactive ·H and ·O species, which corrode the polymer matrix; and high-energy radiation causes fracture or decomposition of organic macromolecular chains. Boxue Du et al. [10] found that introducing fillers capable of generating higher deep-trap energy levels and greater deep-trap densities in polymers can reduce carrier mobility and suppress injected charge transport, thereby mitigating molecular-chain degradation. The incorporation of inorganic nanofillers has shown significant potential for improving the corona resistance of polymer materials [11,12,13]. Qingguo Chen et al. [14] investigated nano-MMT-modified insulating pressboard using thermally stimulated current (TSC) and pulsed electro-acoustic (PEA) methods and reported that MMT filling increased the trap density with increasing filler concentration. The charges captured at the interfaces formed shielding layers, which reduced charge injection from the electrodes and inhibited space charge accumulation. Yanli Liu et al. [15] demonstrated that compared with SiC/EP composites, O-MMT/SiC/EP composites exhibited a denser matrix and fewer voids after thermo-oxidative aging, and the lamellar O-MMT could hinder the outward diffusion of aging products and the inward penetration of oxygen. Ming Ren et al. [16] prepared polyimide films coated with montmorillonite (MMT) nanosheets and evaluated their corona resistance using breakdown as the failure criterion. Their results showed that coating MMT nanosheets on the surface of polymer materials could suppress partial discharge under an electric field and prolong the failure time. Jiao Xiang et al. [17] investigated the corona resistance of EP and EP/SiO_2_ nanocomposites by using AC breakdown strength and surface morphology evolution as evaluation criteria while characterizing trap properties through isothermal surface potential decay (ISPD). Their results indicated that EP/SiO_2_ nanocomposites possessed deeper traps, making charge injection and migration more difficult and thereby enhancing corona resistance.

Overall, substantial progress has been made in improving the corona resistance of insulating nanocomposites and elucidating the associated mechanisms. However, systematic studies on the corona-aging resistance of SiC-based semiconductive anti-corona materials specifically designed for the end regions of large-generator stator bars remain relatively limited. OMMT has potential application prospects for enhancing the corona resistance of semiconductive anti-corona materials by introducing a lamellar structure and increasing trap energy levels and trap density.

In this study, hydrogenated bisphenol A epoxy resin was selected as the matrix material, and nano-OMMT was used as the modifying filler. The effects of the matrix material and OMMT doping on the corona-aging resistance of micro–nanocomposite anti-corona materials were systematically investigated, along with the corresponding mechanisms. This work aims to provide a material-level solution for the long-term reliable operation of anti-corona systems at the end regions of large-generator stator bars.

## 2. Materials and Methods

### 2.1. Materials

In this study, anti-corona materials based on bisphenol A epoxy resin (EP) and hydrogenated bisphenol A epoxy resin (H-EP) were prepared. On the basis of the H-EP-based composite, nano-scale organically modified montmorillonite (OMMT) was further incorporated at mass fractions of 0.5 wt%, 1 wt%, 2 wt%, 3 wt%, and 4 wt%. The OMMT used in this work had a particle size of 5 μm and an interlayer spacing of 2.6 nm. Detailed information on the materials is provided in Table 1.

### 2.2. Sample Preparation

**Figure 1:**
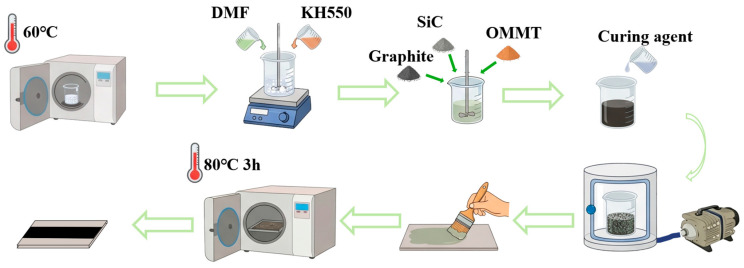
Sample preparation process.

The fillers were dried in an oven at 80 °C for 24 h, and the epoxy monomers were preheated at 60 °C to improve their fluidity. The resin, solvent (DMF), and coupling agent were first mixed under stirring. Subsequently, graphite, OMMT, and SiC were added in batches, followed by mechanical stirring at 300 r/min for 30 min until a homogeneous slurry was obtained. After the curing agent was added, the mixture was vacuum-degassed and then uniformly coated onto the surface of mica plates with dimensions of 80 mm × 135 mm. The coated samples were cured in an oven at 80 °C for 3 h. Finally, specimens with a coating thickness of approximately 0.2 mm were selected for subsequent testing. The sample preparation process is shown in Figure 1.

The anti-corona material prepared from SiC and EP was designated as P4H0, the material prepared from SiC and H-EP was designated as P0H4, and the material prepared from SiC, H-EP, and OMMT was designated as P0H4-OMMT. The mass ratio of SiC to the epoxy matrix was 1:1, corresponding to approximately 42% of the total mass of the composite. The detailed compositions of the samples are listed in Table 2.

### 2.3. Experimental Methods

#### 2.3.1. SEM Microstructural Characterization Testing

A ZEISS Sigma 360 scanning electron microscope (SEM, ZEISS, Oberkochen, Germany) was used in this study to characterize the damage induced by corona discharge on the anti-corona layer of motor stator bars, as well as the dispersion of SiC and OMMT within the matrix of the anti-corona material.

#### 2.3.2. Corona Aging Test

**Figure 2:**
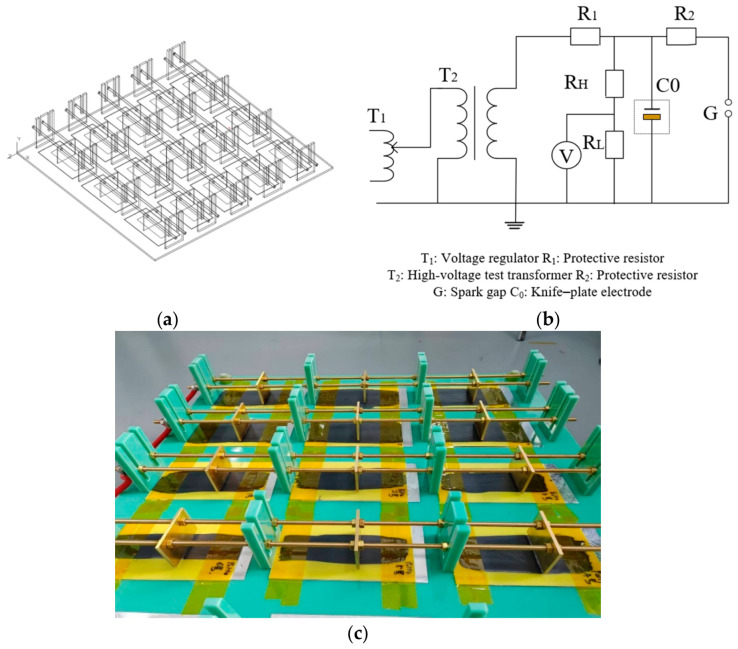
Corona resistance test system (**a**) Test setup design diagram. (**b**) Test setup circuit diagram (**c**) Product image.

With reference to IEEE Std 1799-2012 [18], a multi-sample parallel corona-aging test setup was designed in this study. Its structural design, circuit schematic, and physical configuration are shown in Figure 2.

The test was conducted in a gap-discharge mode. The high-voltage excitation was supplied by an AC high-voltage test transformer, and corona discharge was generated using a knife–plate electrode system. The vertical distance between each knife-edge electrode and the sample surface was set to approximately 2 mm. The test parameters were set as follows: an AC voltage frequency of 50 Hz and an RMS voltage of 5 kV. Aging durations of 24 h, 48 h, 72 h, 96 h, and 120 h were selected.

**Figure 3:**
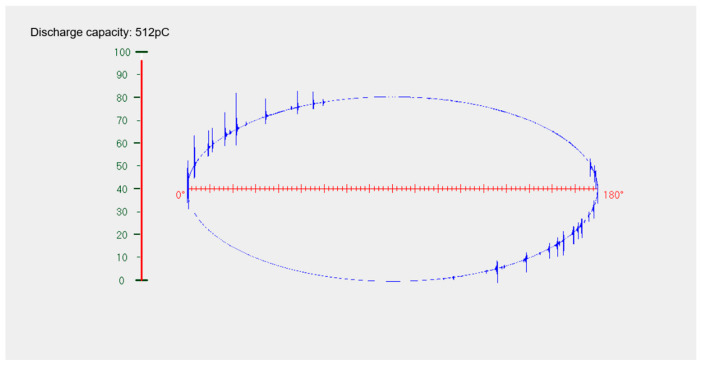
Discharge magnitude during one sinusoidal cycle.

A TCD-9032 (Yangzhou Ruipu Actuator Manufacturing Co., Ltd., Yangzhou, Jiangsu, China) partial discharge tester was used to measure the partial discharge magnitude. The distance between the knife-edge electrode and the sample surface was finely adjusted to ensure that the discharge magnitudes of the knife-edge electrodes were approximately consistent, allowing all samples to undergo corona aging under the same discharge conditions. The discharge magnitude during one sinusoidal cycle for a single knife-edge electrode is shown in Figure 3, with a value of 512 pC.

#### 2.3.3. Polarizing Light Microscopy (PLM) Test

A Leica Visoria P polarized light microscope (PLM, Leica, Wetzlar, Germany) was used to characterize the damage evolution of the anti-corona material during corona aging.

#### 2.3.4. Surface Conductivity Test

**Figure 4:**
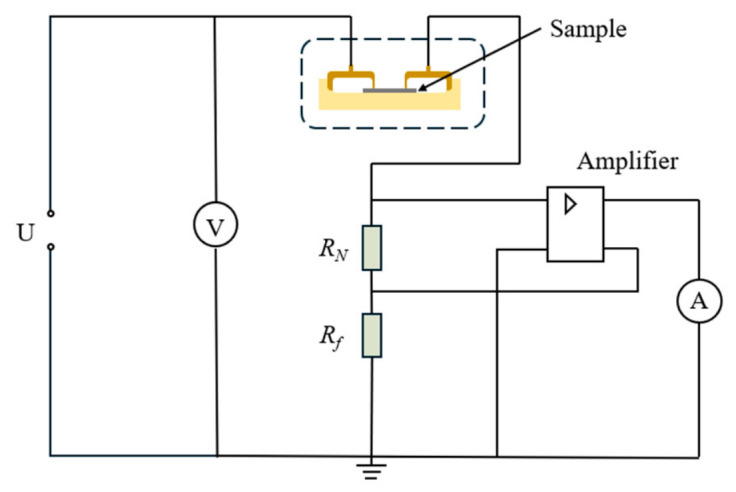
Principle diagram of surface conductivity measurement.

To investigate the evolution of the surface conductive characteristics of the composites under corona aging conditions, a two-electrode system was used to measure the surface conductivity of the composites before and after corona aging. The test setup is shown in Figure 4.

The surface conductivity was calculated using Equation (1),

(1) $$\sigma =\frac{K}{E}=\frac{Id}{lU}$$

where *σ* is the electrical conductivity of the specimen, in S; *K* is the current density, in A/mm; *I* is the measured leakage current, in A; *U* is the input voltage, in kV; *d* is the distance between the electrodes, in mm; *E* is the test electric field, in kV/mm; and *l* is the length of the electrode, in mm.

Nonlinear electrical conductivity refers to the property in which a material’s electrical conductivity increases nonlinearly as the electric field strength increases. The nonlinear characteristics of composite materials are expressed as Equation (2),

(2) $$\sigma =\sigma_{0}\cdot \exp(\beta E)$$

Taking the logarithm of both sides of Equation (2) and simplifying yields Equation (3),

(3) $$\ln\sigma =\ln\sigma_{0}+\beta E$$

where *σ_0_* is the initial surface conductivity, in S, and *β* is the conductivity nonlinearity coefficient, in cm/kV.

The rate of change of surface conductivity is expressed as Equation (4),

(4) $$r=\frac{\sigma_{y}-\sigma_{x}}{\sigma_{x}}\times 100\%$$

where *σ* is the surface conductivity.

#### 2.3.5. Fourier-Transform Infrared Spectroscopy (FTIR)

A Thermo Fisher Scientific Nicolet iS20 Fourier-transform infrared spectrometer (FTIR, USA) was used in this study to characterize the changes in surface functional groups of the anti-corona materials before and after corona aging.

#### 2.3.6. Isothermal Surface Potential Decay (ISPD)

**Figure 5:**
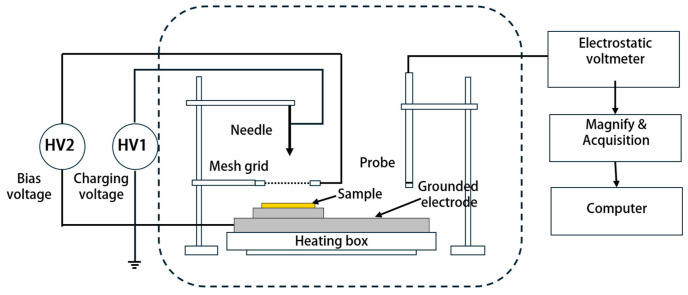
Schematic diagram of the isothermal surface potential decay (ISPD) test setup.

An isothermal surface potential decay (ISPD) test system was used to measure the variation in the surface potential of the samples, from which the trap energy levels and trap densities of the materials were fitted and calculated [19]. The samples were first charged using a corona triode and then transferred to an electrostatic voltmeter for ISPD measurement. The corona triode consisted of a needle electrode, a grid electrode, and a grounded plane electrode. DC high voltages of −5 kV and −10 kV were applied to the needle electrode and the grid electrode, respectively. The electric field between the grid and the grounded electrode was maintained relatively uniform; therefore, charged particles could be uniformly deposited on the sample surface. The initial surface potential was determined by the grid voltage under steady-state conditions. The temperature of the heating stage was set to 30 °C. After charging for 3 min, the sample was transferred to the Kelvin probe of the electrostatic voltmeter within 1 s using a rotatable platform, and the distance between the probe and the sample surface was maintained at 2 mm. The schematic diagram of the test setup is shown in Figure 5.

## 3. Results and Discussion

### 3.1. Dispersion of Fillers in the Anti-Corona Materials

**Figure 6:**
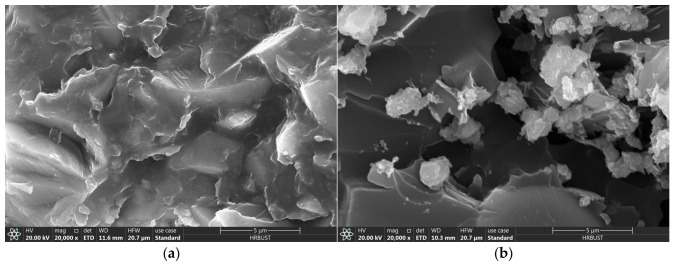
Cross-sectional structures of the anti-corona materials. (**a**) P0H4 and (**b**) 1 wt% OMMT.

Figure 6 shows the cross-sectional SEM images of the materials. As shown in Figure 6a, the SiC particles are uniformly dispersed in the matrix of P0H4. As shown in Figure 6b, in the OMMT-containing P0H4-OMMT composite, OMMT exhibits a lamellar morphology and is uniformly dispersed in the epoxy matrix.

### 3.2. Failure Mechanisms of Anti-Corona Materials in Motors Under Corona Conditions

**Figure 7:**
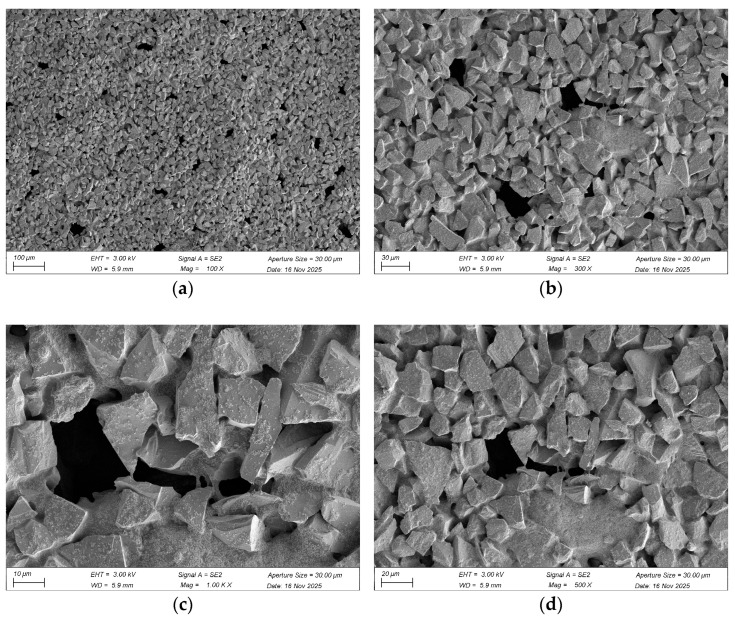
SEM images of the surface morphology of the generator anti-corona material before corona discharge: (**a**) 100 μm; (**b**) 30 μm; (**c**) 20 μm; (**d**) 10 μm.

To elucidate the effect of corona aging on motor anti-corona materials, the surface micromorphology of the anti-corona materials before and after corona aging was observed, as shown in Figure 7.

The SiC particles in the anti-corona material before corona discharge exhibit a distinctly irregular and polygonal morphology. For composites containing irregularly shaped fillers, two contact modes may exist between filler particles, namely face-to-face contact and edge-to-edge contact. This irregular morphology indicates that SiC particles are more likely to form a complex overlapping network within the epoxy resin matrix [20]. As shown in Figure 7a, the SiC particles are densely distributed in the epoxy resin matrix and are connected end to end, forming a highly continuous conductive network. Consequently, the current tends to flow along the shortest conductive path [21].

As observed from Figure 7b–d, many SiC particles are not in complete direct contact with one another but are separated by an extremely thin epoxy resin layer. When the applied electric field is sufficiently high, the resistivity of the nonlinear filler decreases, causing this thin insulating matrix layer to withstand a very high local electric-field strength. This may induce local breakdown of the thin matrix layer and activate the conductive pathway, resulting in a sharp increase in current. The nonlinear behavior of the anti-corona material originates from this mechanism. With increasing electric-field strength, the percolation effect among SiC particles becomes more pronounced, leading to an increase in the conductivity of the anti-corona material and thereby enabling adaptive homogenization of the tangential electric field.

**Figure 8:**
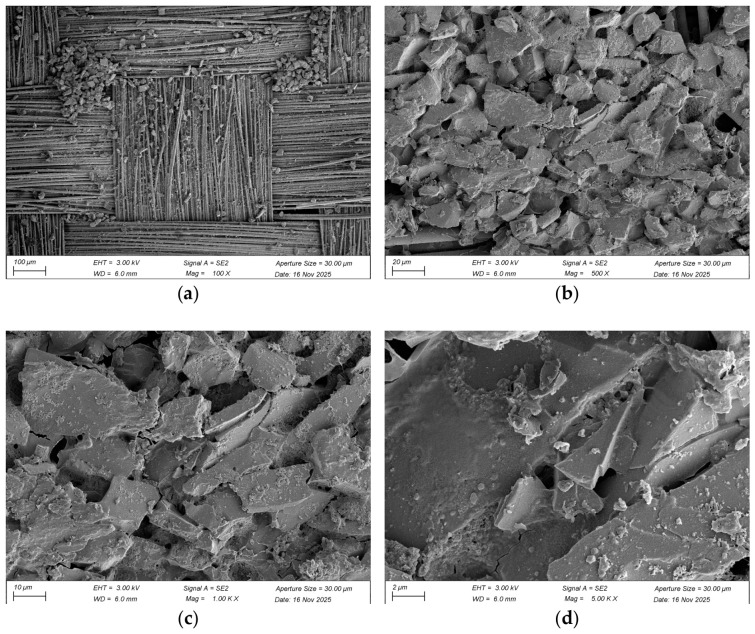
SEM morphology of the anti-corona layer surface after aging: (**a**) 100 μm; (**b**) 20 μm; (**c**) 10 μm; (**d**) 2 μm.

To further reveal the damage process of corona discharge on the anti-corona material, the surface micromorphology of the motor anti-corona material subjected to corona discharge was observed, as shown in Figure 8. Under long-term corona discharge, the epoxy matrix that anchors the SiC particles gradually undergoes aging-induced degradation and cracking. Eventually, the SiC particles detach as a result of complete decomposition of the epoxy resin, leading to the loss of the nonlinear characteristics of the material.

In summary, the functionality of anti-corona materials primarily stems from the conductive pathways formed by the SiC particles and the epoxy matrix. Corona discharge gradually erodes the epoxy matrix, causing the SiC particles to detach and the conductive network to break, thereby altering the current-distribution characteristics of the anti-corona layer. The epoxy matrix plays a decisive role in the corona aging process; improving the matrix’s inherent corona resistance is the fundamental approach to preventing the degradation of the anti-corona layer’s functionality.

**Figure 9:**
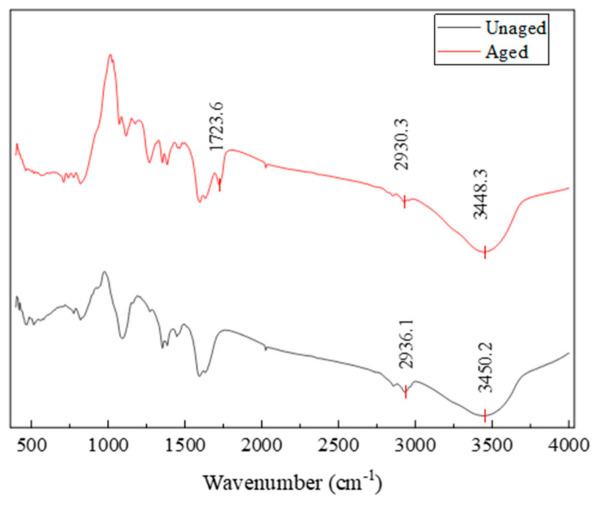
Comparison of FTIR spectra of the anti-corona materials before and after aging.

FTIR analysis was performed on the anti-corona materials before and after aging, and the results are shown in Figure 9.

In the region of 3400–3500 cm^−1^, the unaged material exhibits a broad absorption band, which is generally assigned to the stretching vibration of -OH groups. After aging, the absorption band of the aged sample in this region becomes significantly broadened, accompanied by a relative change in intensity. This indicates that, under high-energy electron bombardment and the ozone-containing environment generated by corona discharge, severe oxidative degradation occurs in the polymer matrix, producing more oxygen-containing polar groups such as hydroxyl and carboxyl groups. In addition, the increased hydrophilicity of the aged material surface may promote the adsorption of water molecules, thereby enhancing the absorption intensity in this region.

The aged spectrum also shows characteristic absorption peaks near 2920 cm^−1^ and 2850 cm^−1^, corresponding to the asymmetric and symmetric stretching vibrations of C-H bonds in methylene (-CH_2_-) or methyl (-CH_3_) groups. After aging, the relative intensities of these peaks decrease and their peak edges become less distinct. This suggests that high-energy particle bombardment during corona discharge causes cleavage of polymer side chains or main chains. Hydrogen atoms in the organic skeleton may be abstracted or oxidized, leading to degradation of aliphatic chain segments and the release of volatile small molecules.

After aging, changes are also observed in the spectral region of 1600–1750 cm^−1^. This region is generally associated with aromatic skeleton vibrations, such as the characteristic benzene-ring peaks in bisphenol A structures, as well as the stretching vibration of carbonyl groups (C=O). The weakening of the aromatic characteristic peaks indicates damage to the macromolecular backbone, while the appearance of broad new absorption bands or peak shifts in this region is typical evidence of oxidation. In the corona environment, ozone and oxygen radicals can readily attack weak bonds in the polymer, inducing ring-opening or chain-scission oxidation reactions and generating degradation products such as ketones, aldehydes, and carboxylic acids.

In summary, the epoxy resin matrix plays a decisive role during corona aging. Therefore, improving the intrinsic corona resistance of the matrix is an important approach to suppressing the functional degradation of anti-corona materials.

### 3.3. Effect of Corona Aging on the Performance of Anti-Corona Materials for Different Substrates

Study [22] indicates that the phenyl/cyclohexyl ratio in epoxy resin monomers significantly affects the corona resistance and insulation properties of EP. As the cyclohexyl content in EP increases, the corona resistance lifetime of EP gradually increases.

#### Effects of Surface Conductance and Nonlinearity Coefficients

**Figure 10:**
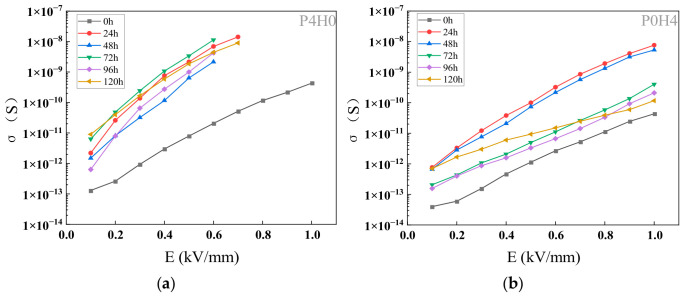
Effect of corona aging on surface conductivity. (**a**) P4H0; (**b**) P0H4.

**Figure 11:**
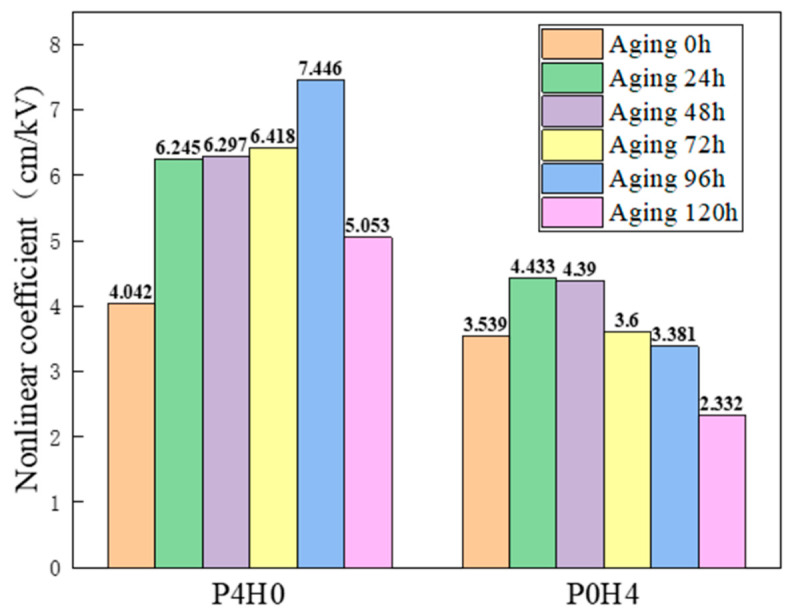
Effect of corona aging on surface conductivity.

Corona-aging tests were performed on the conventional anti-corona material with EP as the matrix and the modified anti-corona material with H-EP as the matrix. The evolution of the surface conductivity and nonlinear coefficient of the samples after different corona-aging durations was investigated, and the results are shown in Figure 10 and Figure 11.

As shown in Figure 10, both P4H0 and P0H4 maintain nonlinear conductive characteristics at all aging stages; however, their conductivity evolution behaviors differ significantly. With increasing corona-aging time, the conductivity curves of P4H0 exhibit an obvious overall upward shift, indicating that, under the same testing electric-field strength, the surface conductivity of the material increases with aging time. When the electric-field strength exceeds 0.65 kV/mm, surface flashover occurs in the samples at all aging stages, resulting in termination of the test. For P0H4, the conductivity curves also shift upward with increasing aging time, but the magnitude of this shift is significantly smaller than that of P4H0. As shown in Figure 11, the nonlinear coefficients of both materials first increase and then decrease. The variation amplitude of the nonlinear coefficient is 3.40 for P4H0 and 2.10 for P0H4.

Based on the comparative results of the two groups of samples, it can be concluded that, during long-term corona aging, the hydrogenated bisphenol A epoxy resin-based anti-corona coating (P0H4) exhibits significantly better conductivity stability and a lower degree of nonlinear-coefficient variation than the bisphenol A epoxy resin-based anti-corona coating (P4H0).

### 3.4. Effect of OMMT Doping on the Electrical Conductivity of Anti-Corona Materials

Both P4H0 and P0H4 exhibit varying degrees of conductivity fluctuation during long-term corona aging. The stability of surface conductivity reflects the ability of the material to resist corona-discharge-induced aging.

#### 3.4.1. Effect of Doping Concentration on Surface Conductivity and Nonlinearity

**Figure 12:**
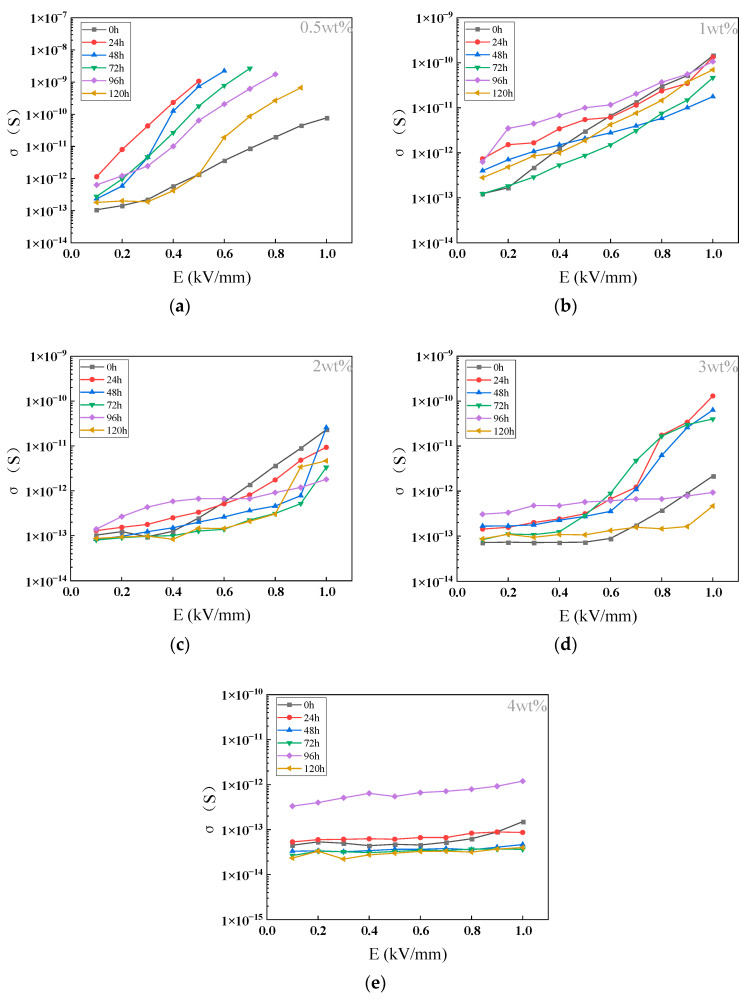
Effect of OMMT doping concentration on surface conductivity. (**a**) Doped with 0.5 wt% OMMT. (**b**) Doped with 1 wt% OMMT. (**c**) Doped with 2 wt% OMMT. (**d**) Doped with 3 wt% OMMT. (**e**) Doped with 4 wt% OMMT.

**Figure 13:**
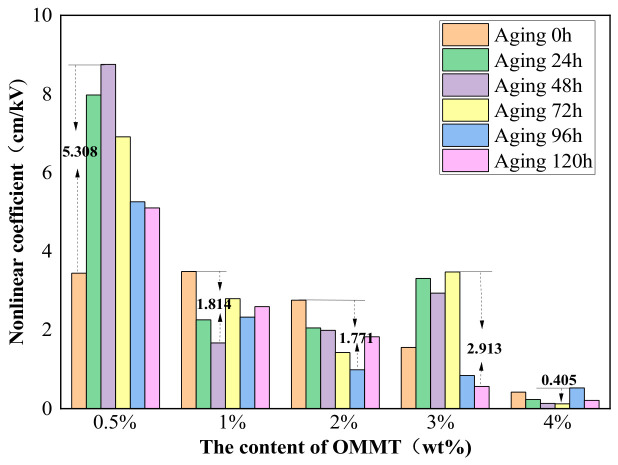
Effect of OMMT doping concentration on the nonlinear coefficient.

Organically modified montmorillonite was introduced into P0H4 at mass fractions of 0.5 wt%, 1 wt%, 2 wt%, 3 wt%, and 4 wt%. The evolution of surface conductivity and the variation in the nonlinear coefficient of the anti-corona coatings during the entire corona-aging period from 0 to 120 h were systematically investigated. The conductivity drift amplitude and nonlinear-coefficient stability were used as the main evaluation criteria, and the results are shown in Figure 12 and Figure 13.

As shown in Figure 12 and Figure 13, the sample containing 0.5 wt% OMMT exhibits large fluctuations in both conductivity and nonlinear behavior throughout the entire corona-aging period. This phenomenon indicates that an OMMT content of 0.5 wt% is insufficient for the nanosheets to form an effective continuous physical barrier network within the matrix. Under continuous corona erosion, local defects rapidly accumulate, resulting in rapid deterioration of the tangential conductive performance and severe instability of the nonlinear coefficient.

**Figure 14:**
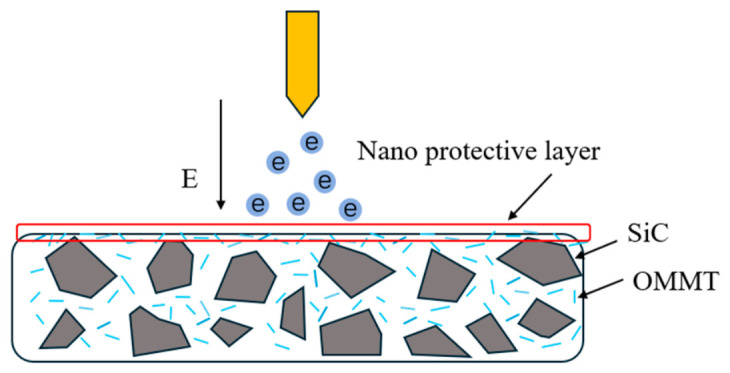
Nano OMMT Protective Layer.

**Figure 15:**
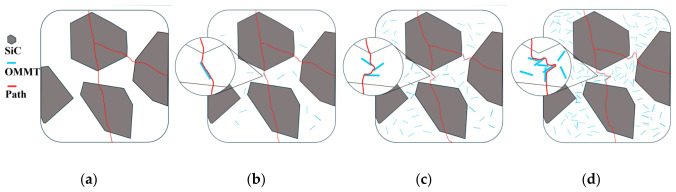
Schematic diagram of the conductive pathway of micro- and nanocomposites. (**a**) Undoped. (**b**) Lightly doped. (**c**) Moderately doped. (**d**) Over-doped.

For the sample containing 1 wt% OMMT, the degree of conductivity drift remains relatively small throughout the aging period, and the nonlinear conductive characteristics satisfy the requirements of practical operating conditions. As shown in Figure 14 and Figure 15, an appropriate amount of OMMT nanosheets is uniformly dispersed in the matrix, forming a physical barrier that effectively suppresses corona-induced erosion of the epoxy matrix. Meanwhile, it does not significantly interfere with the conductive pathways between SiC particles. As a result, a favorable balance between conductivity stability and nonlinear stability is achieved.

When the OMMT content reaches 2 wt%, the interfering effect of the OMMT nanosheets on the conductive pathways between SiC particles becomes evident, and the nonlinear characteristics of the material begin to deteriorate. As the OMMT content continues to increase, the degree of conductivity drift of the anti-corona material gradually decreases; however, the nonlinear coefficient also decreases progressively. When the OMMT content increases to 4 wt%, a large number of OMMT nanosheets are inserted between SiC particles. This physically blocks carrier migration pathways and reduces the overall conductivity of the material to a level at which effective electric-field grading can no longer be achieved. As a result, the anti-corona material completely loses its adaptive regulation capability for the tangential electric field and no longer satisfies the basic functional requirements of anti-corona materials.

Considering conductivity compatibility, conductivity stability, and nonlinear-coefficient stability, the 1 wt% OMMT doping concentration achieves the optimal overall balance. Its conductivity remains within the effective working range of 10^−13^–10^−11^ S, and it exhibits the lowest conductivity variation rate of 104.76%. Moreover, the nonlinear coefficient fluctuates only slightly throughout the 120 h aging period and remains within an effective range. Therefore, 1 wt% OMMT is identified as the optimal modification concentration that satisfies both the functional requirements and long-term stability requirements of anti-corona materials.

#### 3.4.2. Effect of Surface Topography

**Figure 16:**
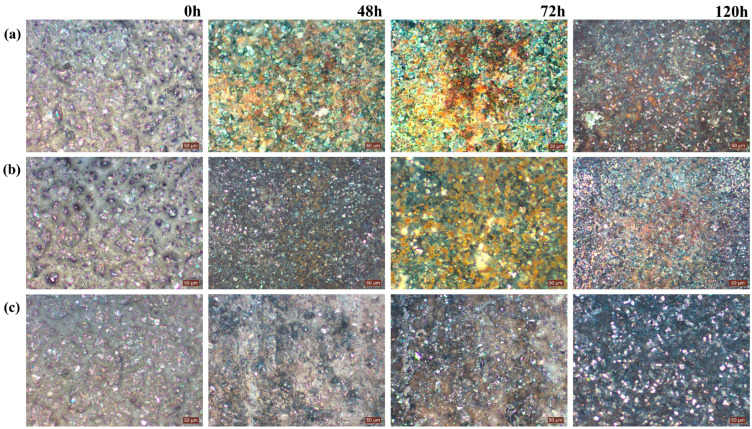
Morphology of anti-corona materials at different aging times. (**a**) P0H4. (**b**) P4H0. (**c**) 1 wt% OMMT.

A polarized light microscope (PLM) was used to observe the surface morphology of P0H4, P4H0, and OMMT-doped samples after different corona-aging durations, so as to verify, at the microscopic level, the protective effects of appropriate OMMT doping and matrix optimization on the structural integrity of the anti-corona materials. The results are shown in Figure 16.

As shown in Figure 16, with increasing corona-aging time, yellowing appears on the epoxy resin surface of the P4H0 sample. Continuous corona erosion further aggravates the yellowing of the epoxy surface, and the eroded region of the matrix gradually expands. After 120 h of corona aging, extensive decomposition or carbonization occurs in the surface epoxy resin, eventually leading to large-scale detachment of SiC particles. In contrast, the epoxy resin matrix of the P0H4 sample contains no unsaturated π-bond structures, resulting in a significantly improved resistance of the surface morphology to corona aging. Consequently, the aging process of P0H4 is markedly delayed compared with that of P4H0. For the sample doped with 1 wt% OMMT, the lamellar structure of OMMT forms a nanoscale protective layer, which mitigates corona-induced damage to the matrix. Throughout the entire aging period, no obvious SiC detachment is observed, and only slight surface carbonization occurs. This further confirms, from the perspective of microstructural stability, the excellent conductivity stability of the material at this doping concentration.

#### 3.4.3. Effect of OMMT Content on Trap Characteristics

**Figure 17:**
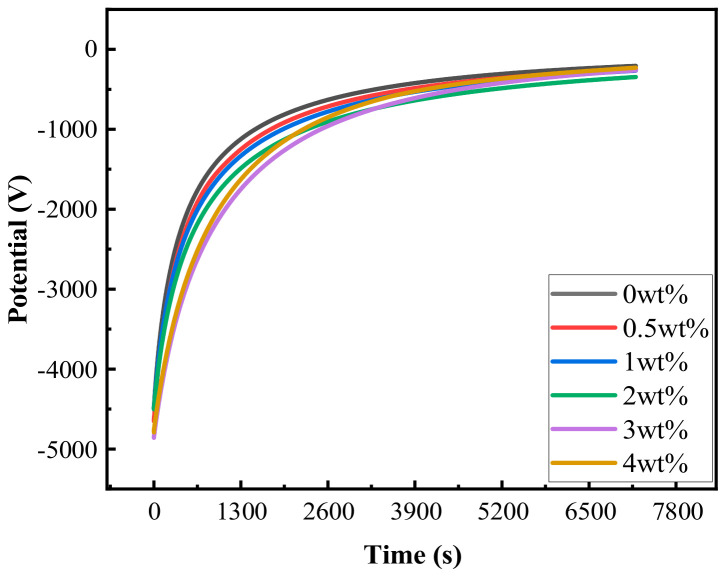
Surface potential decay.

**Figure 18:**
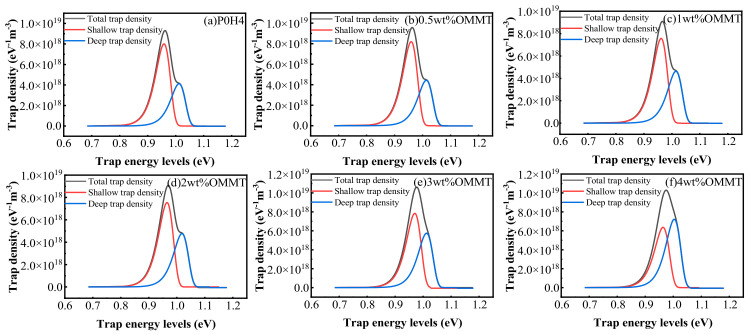
Fitted results of the trap energy level distribution.

**Figure 19:**
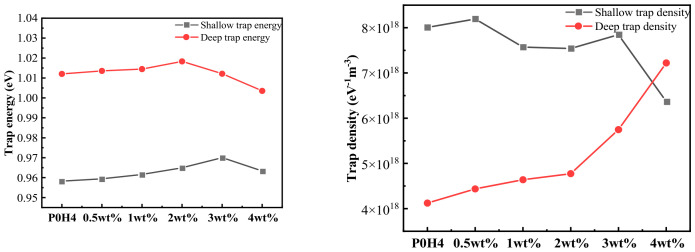
Trap energy levels and trap densities.

From the perspective of trap energy levels and trap density, the regulation of conductivity stability in anti-corona materials by OMMT mainly originates from the numerous micro–nano interfaces formed between its lamellar structure, the epoxy resin matrix, and SiC particles. OMMT possesses a high specific surface area and a lamellar structure. When incorporated into epoxy-based anti-corona coatings, it introduces new interfacial polarization regions and structural defect regions at the OMMT/epoxy, OMMT/SiC, and SiC/epoxy interfaces, thereby altering the trap distribution of carriers within the material. Compared with the undoped system, the incorporation of an appropriate amount of OMMT increases the number of deep traps and shifts the trap energy levels toward deeper states. As a result, captured carriers require higher energy to be detrapped and participate in migration, leading to a reduction in the effective carrier mobility. Consequently, abrupt changes in conductivity caused by injected charges, radical-induced oxidation, and local thermal damage during corona aging are suppressed. The surface potential decay, trap energy level distribution, and variations in trap energy levels and trap densities are shown in Figure 17, Figure 18 and Figure 19, respectively.

In the undoped or low-doped systems, the epoxy matrix is prone to oxidation, chain scission, and local carbonization after corona erosion, resulting in the formation of numerous shallow traps and local defects. These shallow traps have weak carrier-binding capability, allowing carriers to be repeatedly captured and released under the applied electric field. This causes variations in the interfacial barriers between SiC particles and ultimately leads to pronounced fluctuations in surface conductivity and the nonlinear coefficient. When the OMMT content is 0.5 wt%, the number of lamellar structures and the interfacial trap density are insufficient to form an effective trap-regulation network; therefore, the suppression of carrier transport and corona-induced damage is limited.

When the OMMT content is 1 wt%, OMMT is relatively uniformly dispersed in the matrix and can introduce an appropriate amount of deep traps. On the one hand, these deep traps can capture carriers injected or excited during corona discharge, thereby reducing the free-carrier concentration and suppressing local charge migration. On the other hand, an appropriate trap density helps stabilize the interfacial barriers between SiC particles, enabling the material to maintain a relatively stable percolative conductive state before and after aging. Therefore, 1 wt% OMMT can suppress conductivity drift without significantly disrupting the original nonlinear conductive pathways between SiC particles, resulting in superior stability of both surface conductivity and the nonlinear coefficient.

When the OMMT content is further increased, the trap density continues to increase and carrier migration is further restricted. However, excessive OMMT may lead to over-trapping of carriers. Meanwhile, a large number of OMMT nanosheets are inserted between SiC particles, increasing the interparticle distance and barrier width. This weakens electron tunneling, hopping transport, and percolative conduction, thereby reducing the overall conductivity and nonlinear conductive characteristics of the material. Therefore, excessive OMMT is not beneficial for improving the comprehensive performance of the anti-corona material; instead, it weakens its adaptive electric-field grading capability along the surface.

In summary, OMMT regulates the conductivity stability of anti-corona materials mainly by modulating trap energy levels and trap density. Deep traps introduced by an appropriate amount of OMMT can capture carriers, stabilize interfacial barriers, and suppress defect growth induced by corona aging, thereby maintaining the stability of the SiC conductive network. Insufficient OMMT doping provides limited regulation, whereas excessive doping obstructs conductive pathways. Therefore, 1 wt% OMMT achieves an optimal balance between trap regulation and preservation of the SiC conductive network, which is the main reason for the improved conductivity stability of the material.

## 4. Conclusions

This study focuses on the drift of conductive parameters and the degradation of the nonlinear coefficient of SiC-based anti-corona materials used at the end regions of large-generator stator bars under long-term corona exposure. The effects of epoxy matrix type and OMMT content on the corona-aging resistance of the anti-corona materials were systematically investigated. In addition, the failure mechanism and trap-regulation mechanism were analyzed using SEM, FTIR, PLM, surface conductivity measurements, and ISPD tests. The main conclusions are as follows:

The damage induced by corona aging in SiC-based anti-corona materials mainly occurs in the epoxy resin matrix. Corona-induced aging of the epoxy matrix is the primary cause of conductivity drift and the deterioration of nonlinear field-grading capability in the anti-corona layer.

Compared with the bisphenol A epoxy resin-based anti-corona material (P4H0), the hydrogenated bisphenol A epoxy resin-based anti-corona material (P0H4) exhibits better corona-aging stability. Replacing EP with H-EP as the matrix can effectively mitigate corona-induced damage to the epoxy resin matrix and improve the conductivity stability and structural integrity of the anti-corona material.

The OMMT content has a significant influence on the conductive characteristics and nonlinear field-grading capability of the H-EP-based anti-corona material. Among the investigated contents, 1 wt% OMMT is the optimal doping concentration for achieving a balance among conductivity compatibility, conductivity stability, and nonlinear conductive characteristics.

OMMT nanosheets can form a nanoscale protective layer in the matrix, thereby delaying corona-induced erosion of the epoxy matrix. An appropriate amount of OMMT can increase the deep-trap density and trap energy level, enhance the capture of injected carriers, reduce the free-carrier concentration and mobility, and stabilize the interfacial barriers between SiC particles. As a result, conductivity fluctuations during aging are effectively suppressed.

This study provides a new material-design strategy for the long-term reliable operation of anti-corona materials used at the end regions of large-generator stator bars. The temperature sensitivity of the conductivity stability of anti-corona materials remains an important direction for future research.

## Figures and Tables

**Table 1:** Material specifications.

Name	Specifications	Production Manufacturer
SiC	20 µm	Keliansheng New Materials Co., Ltd., Shanghai, China
OMMT	5 µm, Layer spacing: 2.6 nm	Jiangsu Xianfeng Nanomaterials Technology Co., Ltd., Nanjing, China
Graphite	1000 mesh	Henan Liugong Graphite Co., Ltd., Zhengzhou, China
EP	Epoxy value 51	Nantong Xingchen Composite Materials Co., Ltd., Nantong, China
H-EP	Epoxy value 44	Nantong Xingchen Composite Materials Co., Ltd., Nantong, China
1,3BAC	≥99%	Shanghai Macklin Biochemical Co., Ltd., Shanghai, China
N,N-dimethylformamide	1 mol/L	Shanghai Macklin Biochemical Co., Ltd., Shanghai, China
Silane coupling agent KH550	98%	Shanghai Macklin Biochemical Co., Ltd., Shanghai, China

**Table 2:** Sample compositions.

Name	P4H0	P0H4	P0H4-OMMT
SiC/phr	100	100	100
OMMT/wt%	-	-	0.5/1/2/3/4
Graphite/phr	4	4	4
EP/phr	100	-	-
H-EP	-	100	100
1,3BAC/phr	18.5	16.5	16.5
DMF	10	10	10
KH550/phr	5	5	5

## Data Availability

The original contributions presented in this study are included in the article. Further inquiries can be directed to the corresponding author.
